# “精准医疗”理念下单孔VATS肺癌根治术的发展现状、应用细节和展望

**DOI:** 10.3779/j.issn.1009-3419.2016.06.15

**Published:** 2016-06-20

**Authors:** 豫 邓, 志鹏 郝, 向宁 付

**Affiliations:** 430030 武汉，华中科技大学同济医学院附属同济医院胸外科 Department of Thoracic Surgery, TongJi Hospital, TongJi Medical College, Huazhong University of Science and Technology, Wuhan 430030, China

**Keywords:** 单孔电视胸腔镜, 肺癌根治术, 微创胸外科, 精准医疗, Uniportal video assisted thoracic surgery (Uni-VATS), Radical resection of non-small cell lung cancer, Minimally invasive thoracic surgery, Precise medical treatment

## Abstract

单孔电视胸腔镜手术（uni-portal uideo-assisted thoracic surgery, Uni-VATS）的推广，是近年微创胸外科最重大的进展之一。随着腔镜下成像设备、切割缝合器械及电分离器械的改进，单孔VATS的应用范围已从最初的肺组织活检术逐渐扩大到解剖性肺叶/段切除、全肺切除、支气管/血管袖式吻合。多中心大量报道已证实：单孔VATS行肺叶切除安全、可行，清扫纵隔淋巴结的组数及总数均不低于传统多孔VATS，在疼痛、创伤及术后恢复方面也有积极的结果。虽然暂未得到多中心、大样本的临床数据如5年生存率，但有序地逐步开展单孔VATS仍是微创胸外科未来发展的重要方向。本文将围绕单孔VATS的原理及具体操作细节，结合肺癌根治术的基本操作理念进行讨论及综述，以期为单孔VATS的有序、规范化开展提出思考和探索。

## Abstract (en)

Uniportal video-assisted thoracic surgery (Uni-VATS) has become one of the most exciting new developments in minimally invasive thoracic surgery in recent 5 years. With the development of better thoracoscopic cameras and the availability of endoscopic linear mechanical staplers, experts in the technique are able to use uniportal VATS to encompass the most complex procedures such as bronchial sleeve/vascular reconstructions or carinal resections. A large number of multi-center reports have confirmed that Uni-VATS lobectomy is safe and feasible; dissected node group number and total number of mediastinal lymph nodes were not less than that of traditional muli VATS; Uni-VATS gained positive results in pain, trauma and recovery time. Although the large sample multi center clinical data, such as the five year survival rate has not yet been obtained, gradually carring out of uni-VATS will be an important direction for the future development of minimally invasive thoracic surgery. This article will focus on the principle of Uni-VATS and the specific operation details, combined with the basic operation concept, with a view to put forward the orderly and standard utilization of the Uni-VATS.

大量报道已证实：单孔电视胸腔镜手术（Uni-portal video-assisted thoracic surgery, Uni-VATS）行肺叶切除及系统性淋巴结清扫安全、可行，在疼痛、创伤及恢复方面也有积极的结果。很多胸外科中心的Uni-VATS的适用范围已扩展至单孔VATS肺叶切除、肺段切除、全肺切除及双袖式肺叶切除^[1-4]^，并开展了单孔VATS培训计划，充分理解单孔VATs的原理及操作细节，有序地逐步开展单孔VATS是未来发展的重要方向。

本文将结围绕单孔VATS的原理及具体操作细节，结合肺癌根治术的基本操作理念进行讨论及综述，以期为单孔VATS的有序、规范化开展提出一些思考和探索。

## 1 单孔VATS下肺癌根治术的发展现状及面临的问题

2004年单孔VATS用于肺结节活组织检查被Rocco G首次报道^[5]^。近年来，随着头端弯曲的切割缝合器械、分离器械的出现以及光学镜头的精细化，单孔VATS的应用范围在逐渐扩大。2011年Gonzalez-Rivas D首次报道了单孔VATS下肺叶切除术^[6]^，随后大量报道证实，单孔VATS应用于肺叶切除、肺段切除、支气管及血管成型术是安全的、可行的；在术后疼痛控制、术后住院时间及淋巴结清扫数量等方面，单孔VATS亦有较为积极的结果；近期报道的无气管插管条件下^[7]^的单孔VATS肺癌根治术，进一步减少了患者的创伤。单孔VATS下肺癌根治术安全、可行，并取得了较满意的早期结果，已成为近5年来胸外科最重要的进展之一。

但是，针对“单孔VATS能否达到肿瘤的根治性切除并使患者获益”这一核心问题有着不同的声音，这同20年前多孔VATS面临的情形完全一样。在系统性淋巴结清扫的组数及数目上，单孔VATS不低于甚至优于多孔VATS组^[8, 9]^，有较好的预期。但等待最重要的临床数据-5年生存率结果尚需时日。上述问题需要多中心、大样本的临床数据的支持，中小型胸外科单位有序地规范地开展单孔VATs是未来进一步发展的方向。

## 2 手术原理及流程

### 2.1 单孔VATS与传统多孔VATS操作原理的比较与演变

传统多孔VATS向单孔VATS的转变是一个技术、器械及操作原理依次演变并相互影响的过程。传统多孔VATS发展早期的操作原理是：头端笔直的腔镜器械经不同操作孔汇聚于操作面进行顺式操作，操作孔数目、位置及间距决定了手术能否顺利完成。随着技术的精进，术者时常利用观察孔进行操作，使得较多的操作孔失去了意义。但进行单孔VATS时，器械及镜头的相互干扰始终影响操作的顺利进行，器械的改进成为必然。头端可弯曲的腔镜切割缝合器械、电分离及吸引器械的出现，有效回避了器械在单孔内的相互干扰的问题。器械相互交叉后弯曲的头端再次汇聚于操作面进行反式操作，是单孔VATS与传统VATS操作原理的本质不同。

在视角方面，传统多孔VATS镜头和器械经不同切口以不同角度进入胸腔，存在视觉误差^[10]^。单孔VATS器械与镜头经同一切口进入胸腔，视角更接近于直视下开放手术，视觉误差小，已适应多孔VATS视角的术者对单孔VATS视角的适应时间明显大于直接学习单孔VATS的术者^[11-13]^。

在人体工程学角度，传统多孔VATS非直视的视角使术者及助手需随时调整脖颈及背部姿势，单孔VATS在疲劳程度及配合协调度上明显优于传统多孔VATS。

单孔VATS由传统多孔VATS随着技术的熟练、器械的改进演变而来，术者在类似于开放手术的直视视角下以头端弯曲的器械在胸腔内交叉后反式操作，更符合人体工程学。

### 2.2 如何理解普通开胸手术、传统多孔VATS及单孔VATS之间的关系

从微创外科的角度，普通开胸手术、传统多孔VATS及单孔VATS是胸外科手术发展史上三个重要的阶段和手段，无法互相替代。虽然随着器械的革新、技术的精进，单孔VATS的适应指征在逐步扩大，但是，胸外科医师熟练开展单孔VATS则需要有良好的传统多孔VATS以及常规开胸手术基础，这一过程不可逾越。我中心顺利开展的小单孔VATS^[14]^就是在我们前期开展复杂的普通开胸手术^[15, 16]^及精细的传统多孔VATS^[17]^的基础上逐步发展而来的。因此，我中心认为：虽然单孔VATS是微创化发展的方向，但应认识常规开胸手术和传统VATS基本操作原理和技巧的重要性，抛弃“循序渐进”原则开展单孔VATS是危险的。

### 2.3 纵隔淋巴结清扫标准及路径

纵隔淋巴结清扫是肺癌根治术的重点，应包括3站或3站以上纵隔淋巴结（包括隆凸下淋巴结）的清扫，纵隔淋巴结数不得少于10枚^[18]^。

同时，单孔VATS淋巴结清扫也是手术的难点，关键在于淋巴结如何有效的暴露。多数文献报道的各病例系列中淋巴结的组数及总数均达到了清扫要求，但淋巴结暴露及清扫的技巧值得学习。Gonzalez^[11]^强调：手术过程中患者体位应适当变换；应充分松解下肺韧带及肺门结缔组织；镜头始终置于切口上端；辅助器械推挡周围血管、食管及肺组织。但是置入较多辅助器械增加了器械间的干扰，增加了手术难度。来自复旦大学中山医院林宗武等^[8]^报道了患者半俯卧位下的“无抓持整块淋巴结清扫法”，即用吸引器推吸配合下无需抓持淋巴结即能整块切除淋巴结，优势在于省去了抓持器械；半俯卧位有效地暴露了纵隔淋巴结并减少了术者的疲劳程度。但在操作中若遇到伴有机化的或与周围组织粘连紧密的淋巴结则仍需借助抓持器械。

上述单孔VATS纵隔淋巴结清扫下的常用技巧和细节较为实用。但在纵隔淋巴结清扫的路径上，现有文献多遵循"先肺叶/段切除，后淋巴结清扫"路径。该路径下肺叶切除后失去了有效的牵拉，导致淋巴结难以有效暴露；反复摩擦支气管或血管残端，手术风险大大增加（图 1）。

**1:**
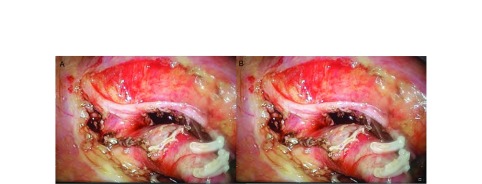
遵循“先肺叶/段切除，后淋巴结清扫”路径，纵隔淋巴结难以暴露。A：先完成右下肺叶切除再清扫第7组淋巴结的弊端：失去了对下肺的牵拉，后纵隔暴露困难，下肺静脉、支气管残端容易受到误损伤；B：先完成左上肺叶切除再清扫第5、6组淋巴结的弊端：失去了对上肺的牵拉，第5组淋巴结显露不佳，左肺动脉干容易受到误损伤。 The mediastinal lymph nodes are difficult exposed post lobectomy. A: The defects to dissect No.7 lymph nodes after the right lower lobectomy: the posterior mediastinal is difficult to expose without the of traction on the lower lung, Inferior pulmonary vein and bronchus are easily injuried by mistake; B: The defects to dissect No.5/6 lymph nodes after the left upper lobectomy: the No.5 lymph nodes are difficult to expose without the of traction on the upper lung, left pulmonary artery trunk is easily injuried by mistake.

因此，我中心提出：充分利用对肺组织的牵拉，"先清扫纵隔淋巴结，再完成肺叶/段切除"的路径。具体的，左胸手术遵循：先清扫第8、9、7、10、5、6组淋巴结，后处理肺；右胸手术遵循：先清扫第8、9、10、7组淋巴结，再处理肺，最后清扫第4、2、3组淋巴结。这在目前文献中没有报道，却能更有效地解决淋巴结。

### 2.4 单孔VATS纵隔淋巴结清扫难点、技巧及方法改进

依据纵隔淋巴结周围解剖结构的特点，熟悉和了解单孔VATS下纵隔淋巴结清扫的目标、体位变化、常用器械、暴露方法将大大降低手术风险并增加清扫的彻底性。

•  主要器械^[11]^：腔镜内卵圆钳、弯头腔镜内吸引器、弯头电凝钩、超声刀、淋巴结钳、腔镜内双关节血管钳、吸痰管。

•  牵拉肺组织的意义：遵循“先肺叶/段切除，后淋巴结清扫”路径时，肺组织的牵拉对淋巴结的暴露无特殊意义，但遵循“先淋巴结清扫，后肺叶/段切除”路径时，肺组织的合理牵拉明显有助于淋巴结的暴露。这一点在现有文献报道中并未特别提及。

主要牵拉方法有：松解下肺韧带及第8、9组淋巴结区时钳夹下肺基底段向胸顶牵引；暴露第7组淋巴结区时，钳夹下叶背段向胸骨方向牵引；游离前肺门组织时，钳夹上叶前段、舌段/中叶肺组织向肩胛方向牵引；清扫上纵隔淋巴结时，钳夹上叶尖/后段向膈肌方向牵引。肺气肿、肺大疱等肺组织质量差者，多选无齿卵圆钳轻柔牵拉。

•  第7组淋巴结^[8, 9]^：文献报道的常规路径大致相同，充分分离淋巴结与食管的间隙，显露双侧主支气管及同侧下肺静脉，从同侧下肺静脉与左主支气管相邻处开始分离淋巴结，先分离左主支气管侧后分离心包侧，用超声刀切断隆凸前的支气管动脉，最后用超声刀分离其与右主支气管的间隙。由于经左胸观察第7组淋巴结位于较深面，清扫最困难，充分推挡主动脉及食管是暴露的关键。为更好暴露第7组淋巴结，我中心在左胸手术时行右侧双腔支气管插管，暴露效果满意，但术中可能出现支气管插管移位，需借助纤维支气管镜及时调整。

•  第5、6组淋巴结^[8]^：使用钳夹小纱布的抓钳将肺往后下方推开，于膈神经前方打开上纵隔胸膜，使用吸引器推压同时使用超声刀分离胸腺后方的淋巴结，游离后打开膈神经后方的上纵隔胸膜，于迷走神经表面打开纵隔胸膜。将第6组淋巴结往后推至膈神经后方。使用吸引器挑起5、6组淋巴结推往背侧，分离其与主动脉弓、动脉韧带、肺动脉干的间隙，显露左侧迷走神经及左侧喉返神经。整块切除第5、6组淋巴结后取出。

•  第2R、4R组淋巴结^[8, 9]^：打开奇静脉弓下缘纵隔胸膜，经奇静脉弓下方充分游离气管周淋巴结，注意此处游离越充分，后续清扫步骤越简单；打开奇静脉弓上缘胸膜，沿奇静脉弓上缘、上腔静脉右侧缘、右迷走神经前缘之间形成的三角区域清扫纵隔淋巴结及脂肪组织。期间注意以吸引器推压淋巴结，以电凝钩或超声刀离断并整块切除淋巴结；可考虑以7号丝线悬吊奇经脉弓或以长弯血管钳钳夹牵拉奇静脉弓辅助暴露该区域。

### 2.5 体位的调整

原则上体位应调整到操作面置于较高水平，可避免渗液堆积于操作面；若吸引器的使用频次增加，则会干扰手术的顺利进行。依据手术部位的不同可通过改变体位、充分利用肺的自身重力帮助显露。清扫后肺门及隆突下淋巴结时应采取前俯卧位，前肺门操作时应采取半后仰位，处理上纵隔、主动脉窗淋巴结时应采取头高脚低位。

### 2.6 直线切割缝合器误伤血管的防范措施

解剖性肺叶/段切除的难点在于血管的暴露及离断，单孔VATS下血管误损伤，术者将十分被动，因此，采取有效的防范措施至关重要。Gonzalez-Rivas^[11]^利用软质束带穿过血管旁间隙后牵拉束带，后置入切割缝合器，退出束带，最后完成击发，该法在远端无血管阻隔时发挥了较好的作用，但远端若有血管阻隔，则仍有损伤该远端血管的风险。我中心的"引导法"可以较好回避该缺点，首先，充分游离血管远心端，为直线切割缝合器创造更多空间；同时，长弯血管钳预先反复通过血管后间隙并开闭数次，可确保切割缝合器置入正确间隙；以Fr12尿管作为引导管，引导管尾端套入直线切割缝合器的扁平端，引导管头端置入血管后间隙，引导直线切割缝合器穿过该间隙后夹闭并离断血管；可以通过牵拉改变肺叶方向，选择最佳的角度以适应器械的置入；直线切割缝合器夹闭血管后应放松对肺组织的牵拉，过度的张力易使血管撕裂。充分游离血管周组织间隙，束带或引导管的合理使用能降低血管误损伤的风险（图 2）。血管损伤的预防的理念需伴随胸外科手术的全程，否则手术难以进行。

**2:**
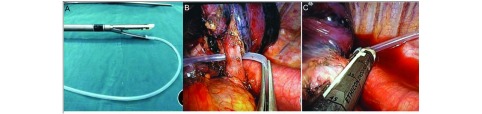
预防血管误损伤的方法。A：选取Fr12尿管作为引导管；B：充分游离血管远心端，改变肺牵拉的方向；C：保持被夹闭组织的适度松弛。 Prevention measures to avoid false injury of blood vessels. A: Choose a Fr12 catheter as the guide tube; B: Full release of blood vessels and change the direction of pulmonary traction; C: To maintain the appropriate relaxation rely on the pulmonary traction.

## 3 切口

单孔VATS在仅有的一个切口完成所有操作，切口位置的选择和切口大小均影响手术进程。

### 3.1 切口的位置

一般来讲，单孔VATS的最佳切口选择在腋前线及腋中线之间的第五肋间，若选第四肋间作手术切口则利于上肺血管的游离，但切割缝合器的角度不佳而无法安全通过肺门组织；若手术切口过低，则肺门结构与器械之间的夹角过小，不利于手术操作；同时，若术中需要中转开胸，则可直接延长手术切口进胸继续手术。对初学者建议严格按照上述方法作切口，但经验丰富后则需依照肿瘤大小及位置决定手术切口位置^[19-21]^。

我中心提出特定手术时需调整手术切口的位置：右中叶切除时切口可选第6肋间，为直线切割缝合器穿过中叶动、静脉及支气管留取了适当的距离；瘦长体型患者行左下肺叶切除时宜选取第六肋间切口，因为清扫第7组淋巴结时往往被左主支气管遮挡视线，增加手术难度，胸廓短小患者可仍选取第五肋间切口。

因此，应结合术者熟练程度、手术目标及肿瘤大小部位等制定切口，以达到治疗的个体化。

### 3.2 切口的大小

由于所有器械均经过同一手术切口，单孔VATS手术切口大小是限制手术难易程度的重要因素之一，同时也是评判手术微创化程度的重要指标。目前文献报道的单孔VATS切口的大小多在3 cm-8 cm之间^[21]^。在手术安全彻底的前提下尽量选取最小的切口开展手术是术者努力方向。

大量文献报道及实践表明^[11, 19, 20]^，充分合理的利用仅有的切口空间理念应贯穿于手术全程。包括：①器械的精细化客观上将更节省操作空间，如直径5 mm的超细腔镜头及未来的无线遥控镜头的使用；②杜绝不必要器械的置入，充分利用吸引器的优势，减少抓持器械的使用；③使用头端弯曲的器械，增加器械的使用效率。我中心利用软质吸引管代替吸引器吸取烟雾，利用体位的变化避免液体堆积于操作面同样可行。我中心单孔VATS肺叶/段切除的切口大小常规为3 cm^[14]^，通过以上“节俭化”原则及器械精细化，手术切口可以小于3 cm，将创伤及疼痛减至更小（图 3）。

**3:**
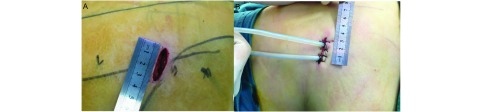
“节俭化”原则下切口大小可以限定在3 cm。A：手术操作中切口的大小；B：手术结束后切口的大小。 Under the principle of "thrifty", the size of the incision can be limited to 3 cm. A: The size of incision between operation; B: The size of incision post-operation.

## 4 器械的选择及原理

### 4.1 电凝钩与超声刀的应用选择

电凝钩和超声刀是常用的腔镜电分离器械，需根据各自特点选取并使用。电凝钩：解剖层次更清晰，全程使用较多，但对支气管动脉等血管止血效果欠佳。超声刀：止血效果好，但是尖端为盲区，有误伤风险（如：大血管、气管膜部、隆突角），常在正常组织界线解剖清晰后再使用，主要应用于离断解剖明确支气管动脉及小血管。在第7组LN隆突夹角处的支气管动脉离断以及10 L淋巴结的清扫时常常使用超声刀。

### 4.2 Echelon与Endo-GIA的选用要点

通常使用的直线切割缝合器包括可弯曲Echelon flex和Endo-GIA。通常选取短钉仓使用，便于直线切割缝合器在胸腔内调整角度。此外，两者的特点不同，Echelon flex需在胸腔外调整角度，通过血管时放松复位通过；而Endo-GIA在胸腔外、内均可调整角度，过血管时仍可随时调整角度。需依据间隙的大小及血管角度合理选取直线切割缝合器。

## 5 术后处理

### 5.1 手术标本的离体方法

标本离体是首先要遵循无瘤及无菌原则，注意保护切口及胸腔^[22]^。离体大标本的取出时可剪取长度约20 cm无菌光缆套封闭一端，置于胸腔上方，袋口朝下，长弯血管钳分别夹持并张开袋口，标本置入后，收紧边绳，取出袋口，再从袋内以小弯血管钳缓慢牵拉出标本，勿暴力牵拉，避免肿瘤播散。

### 5.2 术后胸腔引流管的放置

文献报道手术结束时置24 F胸管1根于切口后方，依层次尤其是深层肌肉组织应严密缝合，皮肤预留丝线备拔除胸管时闭合切口，较粗的胸管可能导致切口愈合不良，将严重影响微创化效果并给患者带来紧张和不便^[19]^。

我中心选取2根更细的16 F胃管，依层关闭手术切口并固定胸腔引流管，术后引流通畅，肺复张良好。双16 F胸管“U”型置管引流能够安全有效地用于单孔胸腔镜肺癌根治患者的术后胸腔引流（图 4），减轻患者疼痛及减少术后引流天数、术后住院天数，降低患者术后三月切口麻木发生率^[23]^。

**4:**
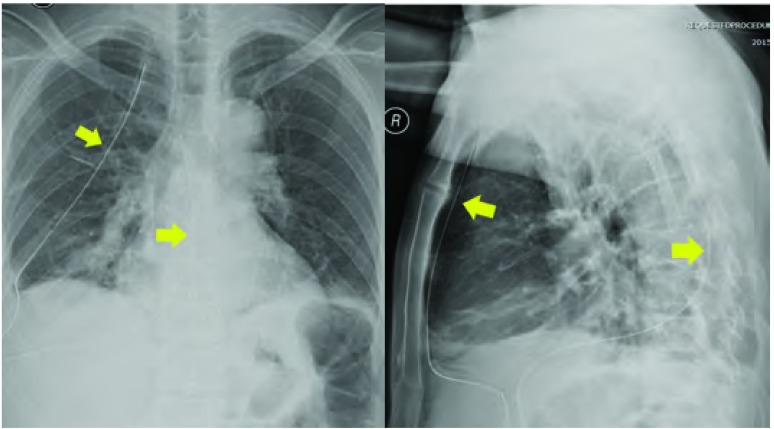
单孔VATS术后留置16 F胸腔闭式引流管，引流效果良好。A：术后胸部正位X线结果；B：术后胸部侧位X线结果。 Chest drainage with two 16 F chest tubes after Uni-VATS got a unobstructed drainage. A: X-ray findings post-operation on thoracic positive position; B: X-ray findings post-operation on thoracic lateral position.

## 6 总结

单孔VATS下解剖性肺叶切除及纵隔淋巴结清扫术已被大量报道证实安全、可行。术后引流量、疼痛控制及术后住院时间均优于传统VATS，纵隔淋巴结清扫组数及数目符合要求，是近5年来胸外科界最重要的进展之一。有序地逐步开展单孔VATS仍是微创胸外科未来发展的重要方向。应在有良好的传统多孔VATS以及常规开胸手术基础上，逐步开展单孔VATS。深入理解单孔VATS操作原理、熟悉具体操作细节，有助于对单孔VATS肺癌根治术的认知和应用，有利于其更好推广，使更多患者获益。
